# Oral hydration is an effective adjuvant treatment for bovine respiratory disease

**DOI:** 10.3389/fvets.2025.1541853

**Published:** 2025-03-28

**Authors:** Júlia Miró, Lorenzo Fraile, Ramon Armengol

**Affiliations:** ^1^Animal Science Department, Universitat de Lleida, Lleida, Spain; ^2^Agrotecnio Research Institute, Universitat de Lleida, Lleida, Spain

**Keywords:** bovine respiratory disease, hydration, treatment, cattle, adjuvant treatment, antimicrobial use

## Abstract

This study aimed to ascertain if oral hydration is an effective adjuvant treatment for bovine respiratory disease. To achieve this objective, clinical and performance outcomes were compared between the calves affected by bovine respiratory disease (BRD) treated with an antibiotic (florfenicol) and a non-steroidal anti-inflammatory drug (NSAID – meloxicam) [group not hydrated (GNH)] and the calves receiving the same antibiotic and NSAID plus an adjuvant therapy based on oral hydration [group hydrated (GH)] at 7% of the body weight (BW) for 5 days. A group of healthy calves, from the same batch and age, was also included as negative control. Crossbred calves (Aberdeen Angus-Holstein Frisian) were enrolled based on a clinical BRD score (0–3 points per clinical sign: rectal temperature, nasal discharge, eye discharge, cough, and ear/head position) during the first 21 days of the fattening period. Thus, a calf was designated BRD-affected if it had a BRD score equal to or higher than 5 points. The BRD-affected calves (*n* = 130) were randomly allocated to the GH (*n* = 65) or GNH (*n* = 65) groups. Clinical score was monitored after 4 days to determine curation or retreatment. Performance outcomes [body weight (BW) and average daily gain (ADG)] were also measured in both experimental groups and in the negative control group at days of inclusion (42 and 80 days) since the beginning of the trial. Throughout the trial, GH showed a significantly (*p* ≤ 0.05) lower clinical score after 4 days and a complete absence of BRD cases becoming chronic compared to GNH. When BW data were analyzed using a multivariable model, considering BW at day 0 as a factor in the model to accurately estimate BW2, BW3, ADG2, ADG3, and ADG global, GH calves showed significantly (*p* ≤ 0.05) higher ADG throughout the trial (at 42 days, 42–80 days, and inclusion–80 days) compared to the GNH ones. In this study, fattening calves with clinical BRD, receiving antibiotic and NSAIDs plus an adjuvant therapy, based on oral hydration at 7% of their BW for 5 days, had better curation rate, less chronic cases, and better performance parameters compared to calves that only received antibiotic and NSAIDs.

## 1 Introduction

Bovine respiratory disease (BRD) is a multifactorial disease distributed among calves worldwide with a negative impact at the productive and economical levels in both fattening and heifer-rearing farms (1). Thus, BRD is the main cause of death between 3 weeks of age until weaning in calves. Despite the interest in improving its control measures, BRD remains the second most frequent disease, after neonatal calf diarrhea in pre-weaned calves (2).

The most common clinical scenario in the development of BRD involves an immunity-depressed calf exposed to a primary infectious agent (usually a viral one), which progresses from the upper respiratory tract (pharynx and trachea) to the lower tract (bronchi to alveoli), inhibiting the activity and function of the ciliated respiratory cells. In this scenario, the innate immune system and the main mucociliary mechanism are compromised (3), allowing bacteria to colonize from the tonsils to the lower respiratory tract, leading to the inflammation and destruction of pneumocytes and the ciliated epithelial cells (4). The importance of the innate immune system in the control of the disease is critical since it is the first defensive barrier against BRD, through the protective intervention of the ciliated epithelial cells of the upper respiratory tract and the secretion of antimicrobials and pro-inflammatory cytokines (5). In this process, the layer of liquid surface (LS) of the upper airways, produced by the submucosal glands and goblet cells, performs an active role in the innate immune defense, altering the structure of bacteria and forming aggregates, which are expelled through ciliary movement and mucus fluidity. The LS layer is basically composed of 97% of water, as well as mucins and proteins with antimicrobial action (4, 6). The calf dehydration may increase the density of the LS layer and reduce the amount produced by the glands, making it difficult to expel the aggregates formed by pathogenic microorganisms and other cellular material from the respiratory tract, predisposing the calf to BRD (7). Because of these pathogenic mechanisms, a serious lung lesion can be developed in the calves. Thus, an appropriate hydration status during a BRD process ensures good mucus production, proper cilia function, and reduced metabolic stress, which could serve as protective factors in the outcome of BRD (6).

BRD is mainly treated with antimicrobials to tackle the bacterial component and with non-steroidal anti-inflammatory drugs (NSAID) to control the inflammatory response (local and systemic), which could play a key role in the clinical progression of the calf (8, 9). Consequently, there is a great interest in improving the diagnosis of BRD to focus on the use of antimicrobials in those calves with pneumonic lesions to accomplish the prudent use of these drugs and to avoid generalized and unnecessary treatments (metaphylactic approaches) to control BRD at the herd level (10). Additionally, the search for adjuvant therapies to improve the curation rates, after applying the “antimicrobial and NSAID approach,” may increase the curation rate, reduce relapses of BRD cases, and optimize the use of antimicrobials (4). One adjuvant therapy could be based on improving hydration in pneumonic calves and avoiding the negative impact that may produce dehydration as an outcome of pneumonia in these animals.

The primary aim of the present study was to evaluate the efficacy of oral hydration containing electrolytes and an energy source (adjuvant therapy) when added to the standard BRD treatment (antibiotic and NSAIDs) compared to the standard treatment without adjuvant therapy in BRD clinical cases. Clinical recovery was primarily assessed as the primary criterion to demonstrate the superiority of the supplemented treatment (with oral hydration) over the standard one (without oral hydration). The secondary criteria were to compare the number of retreatments, relapse episodes, mortality, the incidence of runts, and zootechnical parameters such as average daily gain (ADG).

## 2 Materials and methods

The study protocol was submitted to the Experimental Animal Ethics Committee for Animal Care of the Universitat de Lleida, which approved the trial with the assigned number CEEA 03–02/2.

### 2.1 Farm and animals

The study was carried out in a commercial farm for fattening calves in Maials (Lleida, Northeast Spain) with a herd capacity of 1,000 animals. The study period was scheduled for 1 year, from January 2023 to January 2024. Calves were housed in open-air, straw-bedded boxes in groups of eight during the preweaning period and in groups of 16 at weaning and remained in the farm for 82 days, which was the entire duration of the study. Calves received 450 g of milk replacer (MR) daily (60% sprayed milk, 24% raw protein and 20% raw fat) using a precision scale at a concentration of 12.5% of total solids (as set in the MR manufacturer’s leaflet) for the first 40 days on the farm. Since the first day of arrival, the calves had *ad libitum* access to water, barley hay, and concentrate. Weaning occurred gradually over 7 days, and the calves were completely weaned before they were regrouped. All animals in the study were crossbred Holstein Frisian-Aberdeen Angus male calves that arrived at the farm with an age of 30–40 days of life and an average body weight (BW) of 60 kg. Calves came from dairy farms located close to the fattening farm (30–50 km). At arrival, the history of vaccination and treatments were unknown.

As a standard procedure, all calves received a vitamin complex based on the commercial brand: Complejo B Lamons Carnitine® (L-carnitine + vitamin B12, B1, B2, B6 + folic and nicotinic acid) (Laboratorios Lamons S. A, Lleida, Spain) in 4 g/L of milk for 30 days. Calves were vaccinated and revaccinated 3 weeks later with Bovilis Bovipast RSP® (inactivated bovine respiratory syncytial virus, inactivated parainfluenza virus-3 and inactivated *Mannheimia haemolytica* serotype A) (Merck Sharp & Dohme Animal Health S. L, Carbajosa de la Sagrada, Salamanca, Spain). Finally, at day 70 since arrival, calves were with also vaccinated Bovilis IBR Marker Live® (bovine herpesvirus type 1 strain GK/D) (Merck Sharp & Dohme Animal Health S. L, Carbajosa de la Sagrada, Salamanca, Spain).

### 2.2 Inclusion criteria

Calves were identified by their national identification ear tag number, and they were subjected to a daily visual veterinary inspection. All calves included in this study were additionally monitored twice a week using a calf respiratory scoring chart protocol (11) (Monday and Thursday). Briefly, rectal temperature, cough, nasal discharge, eye discharge and ear position were evaluated and scored following the protocol. As previously described, a global score was obtained by summing the values recorded for the former five parameters, and the calves with a score equal to or greater than 5 were considered a clinical case of BRD. The calf respiratory scoring chart protocol was consistently carried out by the same veterinarian, who was blinded to the treatment group of calves.

### 2.3 Enrollment procedure

**Figure 1:**
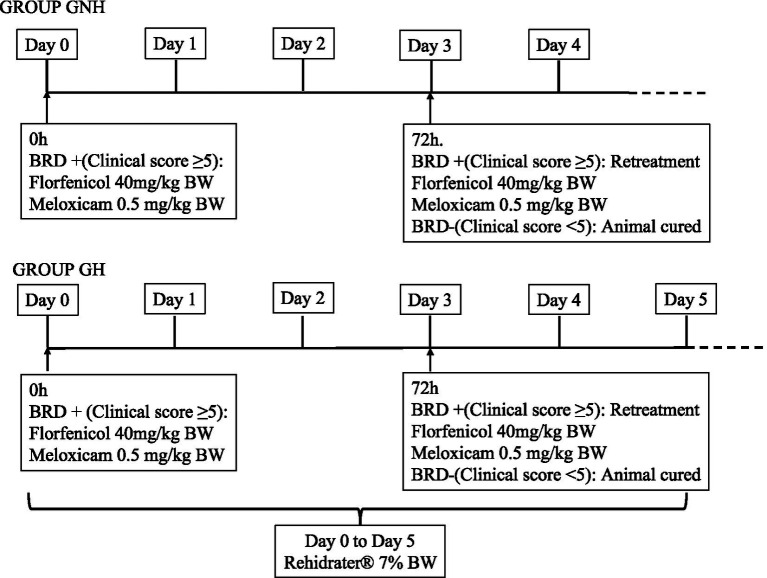
Diagnostic of clinical BRD and treatment for each of the groups under study. GROUP GNH, Group not Hydrated; GROUP GH, Group hydrated; GH: Experimental group composed of clinically sick calves of BRD, treated with florfenicol + meloxicam, and orally hydrated for 5 days in a volume corresponding to 7% of the BW. GNH: Experimental group composed by clinically sick calves of BRD and treated with florfenicol + meloxicam.

Once a clinical case of BRD was diagnosed, it was randomly allocated to one of the two experimental groups [group not hydrated (GNH) or group hydrated (GH)]. Calves in the GNH received a single subcutaneous injection of Zeleris® (400 mg/mL florfenicol +5 mg/mL meloxicam) (CEVA Santé Animale, Bordeaux, France) at a dose of 40 mg florfenicol/kg BW and 0.5 mg meloxicam/kg BW. Calves in the GH received the former treatment but supplemented with oral hydration for 5 days after BRD diagnosis. The oral hydration was carried out by administering 7% of the BW using water and Rehidrater® (glucose 27.8%, sodium chloride 5.3%, potassium phosphate 2.55%) (Chemical Ibérica, Salamanca, Spain) in a proportion of 75 mL of Rehydrater/L of water. The total volume of oral hydration solution was divided into multiple intakes, with a maximum of 3 L per intake throughout the day and administered via suckler and/or tube. In all cases, the same treatment with Zeleris® was repeated after 3 days (72 h) if the calf’s respiratory score remained equal to or greater than 5, subsequent to execution of the calf respiratory scoring protocol, as previously described, to extend the antibiotic treatment. This event was considered as a retreatment for further analysis (Figure 1). Samples in the form of nasopharyngeal swabs from BRD cases were collected from the farm on a quarterly basis as part of the routine commercial process and sent to a laboratory specializing in viral identification, bacterial isolation, and antimicrobial susceptibility testing. Thus, the laboratory (Eurofins Convet S.L.U., 25,005 Lleida, Spain) categorizes the antimicrobial susceptibility of *Pasteurella multocida*, *Mannheimia haemolytica*, and *Histophillus somni* against antibiotics registered to treat these bacteria in cattle as Sensitive (S), Intermediate (I), or Resistant (R) based on clinical breakpoints from the European Committee on Antimicrobial Susceptibility Testing and Clinical and Laboratory Standards Institute (12, 13). Additionally, it considers the category of each antibiotic for its prudent use on farms, following the European recommendation that the proposal of Spanish One Health approach to tackle the antimicrobial resistance (14, 15). Considering the antimicrobial susceptibility results and the legislation about prudent use of antimicrobials in Spain, florfenicol was chosen based on previous results of its antimicrobial susceptibility against BRD pathogens on the farm on a quarterly basis.

Finally, it should be mentioned that a positive control group (calves with BRD that were left untreated) was not included in the study due to ethical and welfare concerns. It was considered inappropriate to leave animals untreated after accomplishing the BRD inclusion criteria due to the high risk of death and unnecessary suffering for the animals. However, the study did include a negative control group, which comprised healthy calves of the same age and group as the sick calves.

### 2.4 Post-enrollment procedures

**Figure 2:**
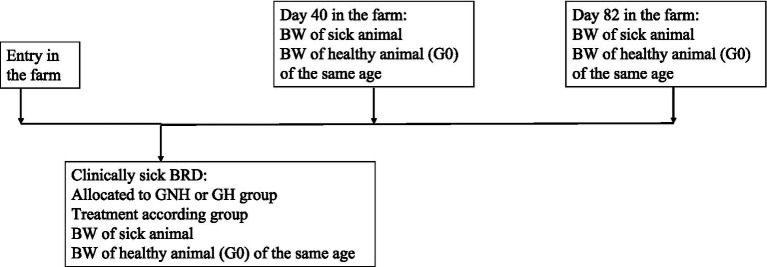
Protocol of BW data compilation along the period for each of the groups under study. Group GNH, Group not hydrated; Group GH, Group hydrated; Group G0, Healthy calves as negative control group; G0: Control group composed of healthy calves; GH: Experimental group composed by clinically sick calves of BRD, treated with florfenicol + meloxicam, and orally hydrated during 5 days in a volume corresponding to 7% of the BW. GNH: Experimental group composed by clinically sick calves of BRD and treated with florfenicol + meloxicam.

On the first day of BRD diagnosis (between 4 and 21 days after entering the fattening facility), the affected calves were weighed (BW1). For each calf diagnosed with BRD included in the study, two healthy calves (Group 0 (G0)) from the same pen, of the same age and number of days after arrival, were also weighed to serve as a negative control. At days 40 and 82 after arrival at the farm, these calves (BRD or healthy) were weighed again (BW2 and BW3, respectively). All weights were estimated using a measuring tape that predicts weight based on the chest circumference. Following a published method (16, 17). In all cases, ADG was calculated as the difference between BW at two control points divided by the number of days between them. Thus, three different ADGs were calculated: ADG from inclusion to day 40 (BW2 to BW1), ADG from day 40 to day 82 (BW3 to BW2), and ADG from the initial day of treatment to day 82 (BW3 to BW1) (Figure 2).

The calf respiratory score was recorded when the calf was included as a clinical BRD case and twice per week to determine if the treatment must be repeated (retreatment). This retreatment must have taken place between 4 and 14 days after the initial treatment to be classified as such. Relapses were defined as cases where a calf that had been previously considered cured of BRD after treatment (respiratory score < 5), later exhibited a respiratory score equal to or greater than 5 at least 15 days after the last treatment. At the end of the study period (82 days of stay on the farm), calves were diagnosed as runts according to the following criteria: The calf was considered not to have recovered if it maintained a respiratory score greater than or equal to 5 after receiving at least four treatments with antibiotics plus NSAID; and/or if it had been separated from the rest of group for an extended period of time; and/or if its BW3 was in the lowest 40 percentile of the batch (3, 10).

The following data were recorded in an Excel sheet to carry out for the data analysis: date, ear tag number, treatment group (Group Not Hydrated (GNH) or Group Hydrated (GH)), pen number, and the score for each of the clinical signs evaluated according to the protocol (rectal temperature, cough, nasal discharge, eye discharge, and ear position). In the same template, other data such as the weight on the first day of BRD, at 40 and 82 days, the retreatments, relapses, and runts were recorded.

### 2.5 Data analysis

All statistical analyses were carried out using SAS V.9.1.3 (SAS Institute Inc., Cary, NC, USA). For all analyses, the individual calf was used as the study unit. The significance level was set at a *p*-value of 0.05. The variables included in the statistical analyses were classified as follows: nominal: treatment group (GNH or GH), retreatment (yes/no), relapses (yes/no), mortality (yes/no), and runts (yes/no); ordinal: number of retreatments; and continuous: clinical score, BW, and ADG. Shapiro Wilk’s and Levene tests were used to evaluate the normality of the distribution of the continuous variables and the homogeneity of variances, respectively. Descriptive statistics were performed for all variables by the treatment group (GNH or GH). Contingency tables (the Chi-squared or Fisher’s exact tests) were used when the association between nominal and ordinal variables was assessed. To study the association between nominal or ordinal variables with the continuous non-normally distributed variables, the Wilcoxon test (with the Mann–Whitney U test to compare each pair of values) was used. To analyze the association between continuous normally distributed variables and nominal or ordinal variables, an analysis of variance (ANOVA) test (with Student’s t-test to compare each pair of values) was used.

In the case of the continuous variables BW and ADG, a stepwise forward selection was performed to build the multivariable model, considering BW1 and treatment group as fixed effects (18). Potential interactions between variables retained in the final model were also included to test for them. Finally, the Tukey–Kramer test was used to perform a pairwise comparison between categories.

The sample size was calculated to be a representative of the number of animals to be included in this research project. Thus, the number of animals needed to detect differences in clinical score with a confidence level of 95%, a statistical power of 80%, and a standard deviation of 1.4 was 65 considering a difference in clinical score of 0.7 (3.3 versus 4) between groups after applying the different treatments.

## 3 Results

A total population of 1,800 calves, divided into four batches, entered the farm during the study period. Among them, 130 calves were diagnosed with clinical BRD and were divided into two groups according to the treatment applied in the calves: GNH = 65 and GH = 65. Additionally, 77 calves were included in the negative control group (G0) (Table 1). In total, data from 207 calves were analyzed in the study.

**Table 1:** Descriptive composition of each group under study.

Total population (*n*)	1800				
Population under study (*n*)	207				
Batches	1	2	3	4	Total
Period in the farm (day/month/year)	29/01/2023–15/05/2023	10/03/2023–02/06/2023	09/09/2023–29/11/2023	01/11/2023–21/01/2024	29/01/2023–21/01/2024
G0	15	31	15	16	77
GH	17	15	17	16	65
GNH	17	16	15	17	65

G0: Control group composed of healthy calves. GH: Experimental group composed by clinically sick calves of BRD, treated with Florfenicol + Meloxicam, and orally hydrated for 5 days in a volume corresponding to 7% of the BW. GNH: Experimental group composed of clinically sick calves of BRD and treated with Florfenicol + Meloxicam.

### 3.1 Clinical outcome

**Figure 3:**
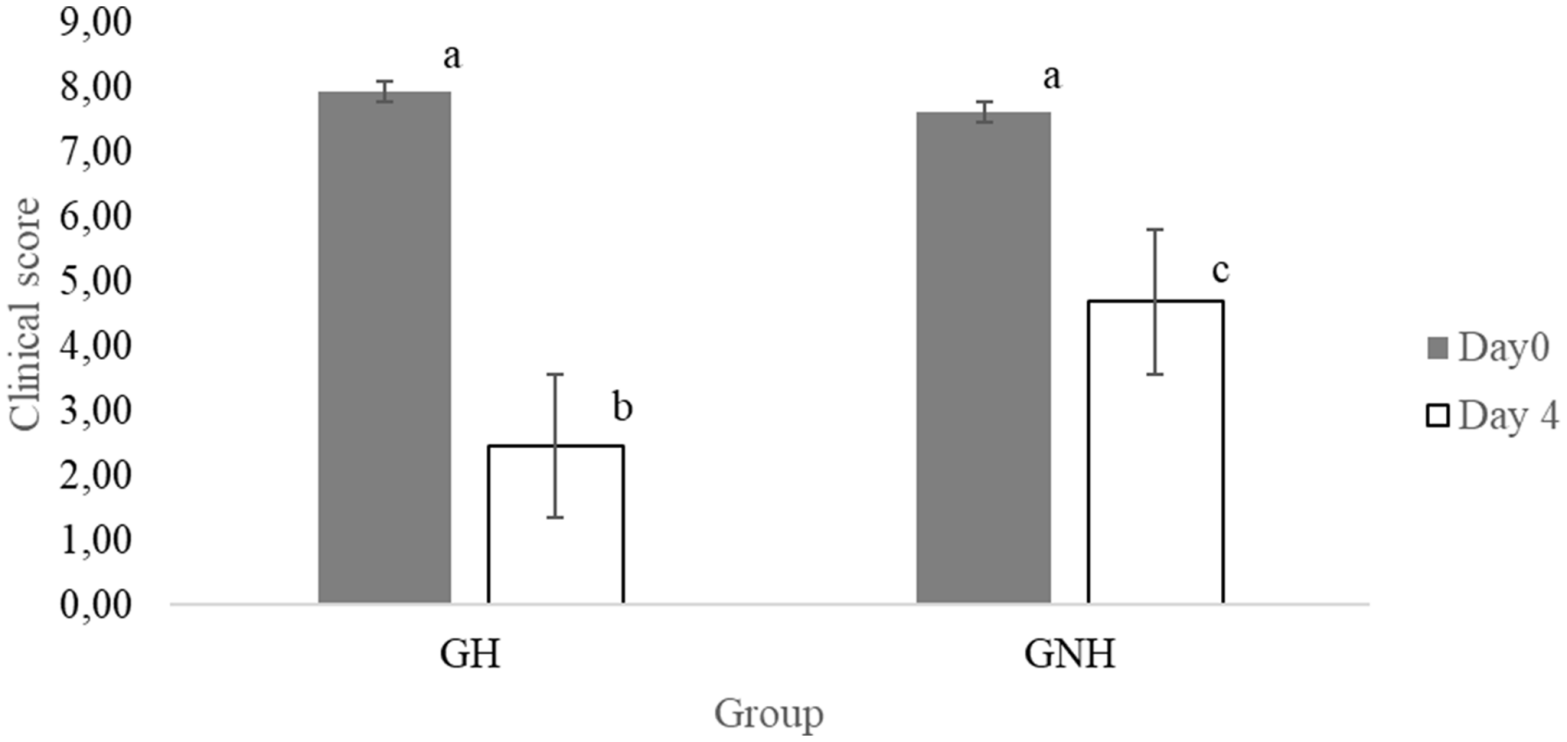
Means (± SE) of the effect of the treatment group on clinical score at the day of inclusion and after 4 days. Different letters between groups mean significant differences between them (*p* < 0.05). GH: Experimental group composed of clinically sick BRD calves, treated with florfenicol + meloxicam, and orally hydrated for 5 days in a volume corresponding to 7% of the BW. GNH: Experimental group composed by clinically sick BRD calves and treated with florfenicol + meloxicam.

The clinical score range among BRD clinically sick calves at inclusion (day 0) was between 5 and 11 points. This score did not show statistically significant differences (*p* > 0.05) between the GNH (7.95 ± 1.59) and the GH (7.89 ± 1.30). However, after 4 days of treatment, the BRD score was significantly higher in the GNH (4.69 ± 2.39) than in the GH (3.15 ± 2.73) (Figure 3). Furthermore, the percentage of calves requiring retreatment (yes/no) showed a statistical tendency (*p* = 0.06) to be higher in the GNH (87.7%) than in the GH (75.4%). Out of the calves that underwent retreatment, 4.6% (6/130), diagnosed as BRD cases, developed chronic disease throughout the entire study, and all of them belonged to the GNH group (6/65, 9.2%). This percentage was significantly higher compared to the percentage observed in the GH group (0.0%) (Table 2). Finally, a total of 19 calves died during the study due to BRD, resulting in a mortality of 14.6% of the BRD cases at inclusion (19/130) because no mortality was observed in the negative control group (GC). The mortality in the GNH (16.9%) was numerically higher than that in the GH (12.3%), but no significant differences were observed between them. All dead calves were submitted to necropsy on the farm. Necropsies were always performed by the same researchers working together in all cases. At farm level, it was determined that the cause of death was of respiratory origin in all cases where only lesions were observed only in the respiratory tract, with no macroscopic lesions in other organs. Briefly, lesions observed in the respiratory tract during the necropsies were mainly located in the cranioventral lung lobes, and they were characterized by bronchopneumonia or its sequelae, including collapse/consolidation, pleural adhesions, abscesses, parenchymal fibrosis, or emphysema. Unfortunately, any organ/tissue sample was sent to the laboratory for further and accurate diagnostics.

**Table 2:** Effect of the treatment group on clinical score, percentage of retreated animals, percentage of chronic cases, and mortality.

	Clinical score day 0 (Mean ± SEM)	Clinical score day 4 (Mean ± SEM)	Retreatment (%) (yes/no)	Chronic cases (%)	Mortality caused by BRD (%)
GH	7.89 ± 1.30	3.15 ± 2.73^a^	75.4^&^	0.0^a^	12.3
GNH	7.95 ± 1.59	4.69 ± 2.39^b^	87.7^&^	9.2^b^	16.9

Different letters between groups indicate significant differences between them (*p* < 0.05). A statistical tendency between groups is represented with “&.” GH: Experimental group composed by clinically sick calves of BRD, treated with florfenicol + meloxicam, and orally hydrated for five days in a volume corresponding to 7% of the BW. GNH: Experimental group composed of clinically sick calves of BRD and treated with florfenicol + meloxicam.

### 3.2 BW and ADG

At the beginning of the study (day 0), there were no significant differences in BW between the GNH (66.1 ± 11.7) and the GH (66.2 ± 12.4) (*p* > 0.05). However, the BW of the negative control group (62.4 ± 10.4) was significantly lower (*p* = 0.04) than that of the GH and was close to being significant (*p* = 0.06) compared to the GNH. Thus, there was no baseline homogeneity at the beginning of the study between the different groups. Therefore, BW at days 40 (BW1) and 82 must be analyzed in a multivariable model, considering BW at day 0 as a factor in the model to correctly estimate BW2, BW3, ADG2, ADG3, and ADG global. After running the model, the goodness of fit (coefficient to determination, R^2^) and the model estimates for the different groups are detailed in Tables 3, 4. BW and ADG were always significantly higher in the healthy calves group (negative control) than in the calves suffering from clinical BRD (GH and GNH) throughout the trial. Moreover, these parameters were also significantly higher in calves receiving adjuvant therapy (oral rehydration (GH)) than in the group without hydration (GNH) throughout the study. Curiously, BRD calves in the GH had an ADG that was higher than that of calves in GNH in 260 g/day, 130 g/day, and 170 g/day during 0–40 days, 40–82 days, and global ADG (0–82 days) period, respectively (Table 4). Global ADG was not statistically associated with the clinical score on the inclusion day (*p* > 0.05).

**Table 3:** LS Means (± SEM) of the effect of the treatment group on body weight (BW) considering BW at day 0 as a fixed effect in a multivariable model.

Body weight (kg)		
	BW2 (R^2^ = 0.60)	BW3 (R^2^ = 0.56)
G0	91.70 ± 1.21^a^	136.7 ± 1.67^a^
GH	81.03 ± 1.37 ^b^	120.5 ± 1.90 ^b^
GNH	76.20 ± 1.39 ^c^	111.5 ± 1.98^c^

Different letters between groups indicate significant differences between them (*p* < 0.05). G0: Control group composed of healthy calves. GH: Experimental group composed by clinically sick calves of BRD, treated with florfenicol + meloxicam, and orally hydrated during 5 days in a volume corresponding to 7% of the BW. GNH: Experimental group composed by clinically sick calves of BRD and treated with florfenicol + meloxicam.

**Table 4:** LS Means (± SEM) of the effect of the treatment group on average daily gain (ADG) considering body weight (BW) at day 0 as a fixed effect in a multivariable model.

Average daily gain (ADG) (g)			
	D40–D0 (ADG2) (R^2^ = 0.36)	D82–40 (ADG3) (R^2^ = 0.18)	D82–D0 (ADG global) (R^2^ = 0.37)
G0	1,110 ± 40 ^a^	1,080 ± 30 ^a^	1,100 ± 30 ^a^
GH	720 ± 50 ^b^	960 ± 40 ^b^	860 ± 30 ^b^
GNH	460 ± 50 ^c^	830 ± 40 ^c^	690 ± 30 ^c^

Different letters between groups indicate significant differences between them (*p* < 0.05). G0: Control group composed of healthy calves. GH: Experimental group composed by clinically sick calves of BRD, treated with florfenicol + meloxicam, and orally hydrated during 5 days in a volume corresponding to 7% of the BW. GNH: Experimental group composed by clinically sick calves of BRD and treated with florfenicol + meloxicam.

## 4 Discussion

Calves suffering from BRD can reduce feed consumption by approximately 77 g/day, and consequently, ADG may be potentially reduced to 67 g/day in comparison with healthy calves (3, 19). It has also been demonstrated that calves dehydrated due to long-distance transportation or those subjected to an extended period of low water consumption for other reasons are predisposed to BRD (7). In the present study, as expected, the group with the best productive performance is that of the healthy calves. This result highlights that control measures to avoid BRD appearance could be very profitable at the farm level. The results suggest that calves receiving oral hydration (GH) have a higher ADG than the ones not receiving it (GNH) as an adjuvant therapy for clinical BRD cases. This result does not agree with the findings of Ref. (20), where no statistically significant differences were observed in daily feed intake and ADG between calves orally hydrated or not at the beginning of the fattening period. However, it should be noted that only 4% of the BW of water was administered—without electrolytes or glucose—to all animals upon entrance, regardless of whether they were suffering from BRD or not. In contrast, our study used rehydration with higher volumes (7% of BW for 5 days), supplemented with electrolytes and glucose, in calves with a robust diagnosis of clinical BRD. This difference in study design could be critical due to the different physiological needs between animals suffering from BRD and those that are not. It is expected that the improvement of mucus and ciliary function in the respiratory system is relevant in BRD-affected calves, which may play a key role in the severity of and recovery from BRD (6). Finally, Ref. (20) studied the effect of hydration in older and heavier animals (BW = 188 kg and older than 100 days of age) compared to our study, where calves were much younger and lighter. Therefore, they could be more susceptible to contracting BRD due to a less mature immune system (21).

Our results also suggest that there is an improvement in the calf clinical score at 4 days post-treatment in the calves that received oral hydration, complementing the antibiotic and NSAID therapy (GH), compared to the ones that only received antibiotic and NSAID treatment (GNH). This effect could be attributed to the increase of the fluidity of the double layer of LS of the upper airway’s mucosa, promoting the expulsion of aggregates of cellular debris, bacteria, and viruses (4). Furthermore, oral hydration directly prevents intrinsic dehydration and weight loss caused by the decrease in feed and water intake, because of fever and toxemia caused by clinical BRD (22). The present study shows that the administration of oral hydration could reduce the appearance of chronic calves in the GH group (0%) compared with the GNH group (9.2% of the calves ended as chronic cases). Unfortunately, no studies have been found in the literature to support the effects of hydration on BRD chronicity. Nevertheless, it can be hypothesized that the adjuvant therapy with Rehidrater® could improve the response of the calves’ innate immune system and enhance the efficacy of the treatment in preventing chronicity. Finally, the adjuvant therapy did not significantly reduce the mortality in this trial. Cattle suffering a severe case of BRD have less protective capacity against the cytotoxic effects of histones, resulting in important and irreversible damage to lung tissue (23, 24). Thus, it could be suggested that oral hydration may not be decisive in severely affected calves, where the antibiotic and NSAID treatment were also ineffective as well. Therefore, the most suitable option in these cases could have been administering intravenous fluid therapy (25) that was not considered in this study.

To the authors’ knowledge, no previous studies have reported that the administration of electrolytes and/or glucose with oral hydration in BRD-affected calves improves their recovery and performance. However, the glucose and electrolytes contained in the oral fluids used in the present study could enhance the effectiveness of the functioning of the cilia, providing the energy needed by the cells of the respiratory tract, and improving the production and quality of the mucus, which is essential for maintaining the physical barrier in the upper respiratory tract. Thus, our results clearly support the addition of oral rehydration therapy containing electrolytes and an energy source as an efficacious adjuvant therapy to the classical BRD treatment approach based on the use of antimicrobials and NSAIDs. This adjuvant therapy reduces the negative impact on ADG due to BRD. This type of supportive therapy could be very beneficial for the farm economy since the return-on-investment (ROI) should be very high due to the low cost of hydration therapy. However, the exact ROI for this therapy should be calculated for a case-by-case situation, since labor cost could vary widely between farms, countries, and/or regions. This adjuvant treatment should be further studied with other age ranges and BWs to increase the external validity of these results and higher statistical power is necessary to study variables as mortality in a more robust and consistent way.

## Data Availability

The raw data supporting the conclusions of this article will be made available by the authors, without undue reservation.
